# Supplementary material for: Having oneself as a stake- a qualitative study on Work-related psychosocial health among emergency call centre operators
Source: Int J Qual Stud Health Well-being. 2025 Dec 4;20(1):2595751. doi: 10.1080/17482631.2025.2595751 (PMC12679843; doi:10.1080/17482631.2025.2595751)
Supplement: Supplementary Material — coreq_word_251123 [file ZQHW_A_2595751_SM8676.docx]

| **Section/Topic** | **Item No** | **Checklist item** | **Reported on page No** |
| --- | --- | --- | --- |
| **Domain 1: Research team and reﬂexivity** | | | |
| Personal Characteristics | | | |
| *Interviewer/facilitator* | 1 | Which author/s conducted the interview or focus group?Interviewer/facilitator | 1,6,19 |
| *Credentials* | 2 | What were the researcher’s credentials? E.g. PhD, MD | 1 |
| *Occupation* | 3 | What was their occupation at the time of the study? | 1 |
| *Gender* | 4 | Was the researcher male or female? | 1 |
| *Experience and training* | 5 | What experience or training did the researcher have? Relationship with participants | 1,6 |
| Relationship with participants | | | |
| *Relationship established* | 6 | Was a relationship established prior to study commencement? | 6 |
| *Participant knowledge of the interviewer* | 7 | What did the participants know about the researcher? e.g. personal goals, reasons for doing the research | 6 |
| *Interviewer characteristics* | 8 | What characteristics were reported about the interviewer/facilitator? e.g. Bias, assumptions, reasons and interests in the research topic | 6 |
| **Domain 2: study design** | | | |
| Theoretical framework | | | |
| *Methodological orientation and*  *Theory* | 9 | What methodological orientation was stated to underpin the study? e.g. grounded theory, discourse analysis, ethnography, phenomenology, content analysis | 5 |
| Participant selection | | | |
| *Sampling* | 10 | How were participants selected? e.g. purposive, convenience, consecutive, snowball | 5 |
| *Method of approach* | 11 | How were participants approached? e.g. face-to-face, telephone, mail, email | 6 |
| *Sample size* | 12 | How many participants were in the study? | 5 |
| *Non-participation* | 13 | How many people refused to participate or dropped out? Reasons? |  |
| *Setting of data collection* | 14 | Where was the data collected? e.g. home, clinic, workplace | 6 |
| *Presence of non-participants* | 15 | Was anyone else present besides the participants and researchers? | 7 |
| *Description of sample* | 16 | What are the important characteristics of the sample? e.g. demographic data, date | 5 |
| Data collection | | | |
| *Interview guide* | 17 | Were questions, prompts, guides provided by the authors? Was it pilot tested? | 6 |
| *Repeat interviews* | 18 | Were repeat interviews carried out? If yes, how many? |  |
| *Audio/visual recording* | 19 | Did the research use audio or visual recording to collect the data? | 6 |
| *Field notes* | 20 | Were ﬁeld notes made during and/or after the interview or focus group? | no |
| *Duration* | 21 | What was the duration of the interviews or focus group? | 6 |
| *Data saturation* | 22 | Was data saturation discussed? | 6 |
| *Transcripts returned* | 23 | Were transcripts returned to participants for comment and/or correction? | no |
| Domain 3: analysis and ﬁndingsz  Data analysis | | | |
| *Number of data coders* | 24 | How many data coders coded the data? | 7 |
| *Description of the coding tree* | 25 | Did authors provide a description of the coding tree? | 7 |
| *Derivation of themes* | 26 | Were themes identiﬁed in advance or derived from the data? | 7 |
| *Software* | 27 | What software, if applicable, was used to manage the data? | 6 |
| *Participant checking* | 28 | Did participants provide feedback on the ﬁndings? | no |
| Reporting | | | |
| *Quotations presented* | 29 | Were participant quotations presented to illustrate the themes / ﬁndings? Was each quotation identiﬁed? e.g. participant number | 8 |
| *Data and ﬁndings consistent* | 30 | Was there consistency between the data presented and the ﬁndings? | 7 |
| *Clarity of major themes* | 31 | Were major themes clearly presented in the ﬁndings? | 7 |
| *Clarity of minor themes* | 32 | Is there a description of diverse cases or discussion of minor themes? | 6 |
