# Supplementary material for: Having oneself as a stake- a qualitative study on Work-related psychosocial health among emergency call centre operators
Source: Int J Qual Stud Health Well-being. 2025 Dec 4;20(1):2595751. doi: 10.1080/17482631.2025.2595751 (PMC12679843; doi:10.1080/17482631.2025.2595751)
Supplement: Supplementary Material — Clean copy Manus_241016 [file ZQHW_A_2595751_SM8677.docx]

**Having oneself as a stake- a qualitative study on**

**Work-related psychosocial health among emergency call centre operators**

ALÉX Jonas^1^ (RN, PEN, PhD) *, GARDFALL Erik^2^ (RN, PEN, MSc), NORDFJÄLL Mikael^2^ (RN, PEN, MSc), MOLIN Jenny^2^ (RN, PhD)

1. Department of Nursing, Umeå University, Division of Ambulance Service, Region of Västerbotten, Umeå, Sweden
2. Department of Nursing, Umeå University, Umeå, Sweden.

**Corresponding author: Jonas Aléx, Department of Nursing, Division of Ambulance Service Region of Västerbotten, Umeå University, SE-901 87, Umeå, E-mail:* [*Jonas.alex@umu.se*](mailto:Jonas.alex@umu.se)

**Abstract**

**Background:** Although emergency call centre operators are not physically present at the scene of emergencies, they are still significantly impacted by these events. They are exposed to distressed individuals who express feelings of fear, desperation, and helplessness. Emergency call centre operators must endure this emotional exposure while simultaneously collecting crucial information from callers, relaying this information to responding units, and potentially instructing callers in critical first aid.

**Motive:** Limited research exists on the work environment and health conditions of emergency call centre operators. The motive of this study is to enhance understanding of the work environment and challenges faced by emergency call operators. The findings are relevant for employers and other professionals within the emergency care chain.

**Aim:** This study aims to describe the experiences of work-related psychosocial health among emergency call centre operators.

**Methods:** This study employed a qualitative approach, with data collected through 16 semi-structured interviews. The analysis was conducted using qualitative content analysis.

**Result:** The analysis revealed one main theme, four themes, and 12 subthemes. The main theme was “Having oneself as a stake,” built on the themes “Facing challenges,” “Being affected as a person,” “Feeling ignored by organisational conditions,” and “Drawing strength from rewarding conditions.”

**Conclusion:** The workplace environment of emergency call centre operators has a negative impact on their psychosocial health. This can lead to decreased quality of emergency call responses and less accurate assessments of critical situations. Further research is needed to identify improvements in operational conditions for emergency call centre operators and establish goals for enhancing work-related health.

**Keywords**: Emergency call centre operators, emergency medical services, psychosocial workplace environment, ambulance, qualitative content analysis.

**Introduction**

Emergency call centre operators (EMCCOs) take distressing calls from frightened, helpless, and desperate callers (1). Although EMCCOs are not physically present at the scene of emergencies, they can still experience these calls as traumatic events (2). They must simultaneously manage critical incidents and be prepared for unforeseen developments during calls. Unfortunately, the understanding of how the conditions and work environment of EMCCOs impact their psychosocial well-being is limited.

**Background**

The Emergency Medical Service (EMS) encompasses healthcare services meticulously designed, staffed, and equipped to provide emergency patient care (3). Within this framework, EMCCOs play a pivotal role: they serve as the first point of contact for callers who dial the national emergency number, connecting them to an emergency call centre. Their significance lies in initiating the chain of care for patients (4).

EMCCOS are often exposed to callers in stressful situations and may feel fear, anxiety, uncertainty, shame, and other strong emotions. EMCCOs must work with people in crisis, identify the type and scope of an emergency, gather critical information, and provide callers with clear instructions (5). The nature of emergency calls varies widely: they may involve incidents where individuals have been victims of or witnesses to crimes, fires, or acute medical conditions, and in these situations, every second counts. EMCCOs face the daunting task of swiftly assessing a situation, aided by system support, to determine what has occurred and where. Callers are often distressed, upset, and shocked, and challenging to extract information from. The demands on EMCCOs are immense; they must skilfully engage with callers to ascertain the appropriate rescue efforts required (6). Despite the crucial nature of their work, EMCCOs face significant stressors. They manage traumatic incidents, such as drownings, shootings, fires, vehicle accidents, and suicides, while being expected to remain calm and composed. Their ability to analyse and transmit critical information during high-pressure situations is essential for determining the emergency status of each call. Consequently, they encounter potentially traumatic events at rates that may exceed those of other first responders (7). Many calls include difficulties relating to the caller, their symptoms, or other circumstances. Language barriers and rude, agitated callers are an increasing problem (8). Additionally, the ability of EMCCOs to recover after work, communicate and collaborate with their colleagues in the workplace, manage their perceived workload, take time for breaks and pauses, and prioritise their work tasks significantly affects work-related psychosocial health (9).

Research across various fields underscores the impact of work-related psychosocial stress on individual well-being and societal dynamics. Small- and medium-sized enterprises grapple with stressors from diverse factors, including work content, task demands, social interactions, and organisational change (10). The connection between workplace accidents and the work environment revealed that an unfavorable work setting can precipitate physical and mental injuries. The consequences of this include poor health, extended sick leave, and challenging reintegration into work. These stressors may even lead to social isolation and financial strain. Significantly, compromised psychosocial health correlates with suboptimal physical health, obesity, and poor mental health, ultimately affecting the quality of emergency call handling (11, 12). In addition, the constant exposure to violence, emergency incidents, graphic details, and frantic encounters with callers puts this population at high risk of problems such as depression, loss of human compassion/empathy, behavioral changes, and decline in cognitive function (13, 14). The work of an EMCCO is not only demanding, but it also presents a high risk for the development of post-traumatic stress disorder (PTSD) (15). Based on the PTSD Checklist–Civilian Version questionnaire, emergency operators in the UK reported a PTSD prevalence of 13–15% (16). EMCCOs experience high levels of both occupational and individual stressors, and more than one-third of EMCCOs report experiencing clinically significant levels of anxiety (35%), depression (35.6%), and PTSD (29.3%). These rates are far higher than the general population's (7). Common operator stressors include the constant risk of a fatal decision-making error, caller frustration, a sense of helplessness, insufficient or no training, and continuous underappreciation(17). Despite being negatively affected by their jobs, many are unwilling to seek help (18). One study found that the top three reasons for not seeking help were: a fear of services not being confidential, that seeking help would have a negative impact on their career, and the possibility of judgment from coworkers and managers (19). In recent years, recruitment and retention levels have fallen, and candidates for EMCCO positions are dropping out before completing their training due to early burnout. Despite their key role, limited attention has been paid to this area of research (20).

**Rationale for the study**

The psychosocial health of EMCCOs is a critical area of concern, as their work exposes them to duty-related trauma, placing them at an increased risk of mental health challenges such as stress, anxiety, depression, and PTSD. Knowledge of the psychosocial health of EMCCOs is low and to our knowledge, no study has been conducted in a Swedish context. Therefore, the motive of this study is to inductively and exploratively contribute to increased knowledge about the work-related psychosocial health of EMCCOs.

**Methods**

**Aim**

This study aims to describe the experiences of work-related psychosocial health among emergency call centre operators.

**Design**

This study used an inductive qualitative design with individual interviews analysed with qualitative content analysis according to Graneheim and Lundman (21).

**Context**

EMCCOs at emergency response centres in Sweden respond to calls to the national emergency number. This involves handling inquiries related to acute injuries and illnesses, and often necessitates deploying prehospital medical resources, including ambulances and/or rescue services. The work does not require any previous training in crisis management or healthcare; instead, EMCCOs are trained internally in connection with their employment. The work takes place in an open space where operators sit side by side and respond to calls while viewing a list of incoming calls waiting to be answered. Among the operators are supervisors and managers who monitor the work. All calls are recorded, and operators receive regular feedback from their managers. During the shift, operators are given strictly regulated breaks of varying lengths.

**Participants**

We employed convenience sampling. Information about the study was sent to EMCCOs via their employer, and those interested in participating in the study contacted the researchers. In total, 16 participants (2 men, 14 women) from emergency call centres in Sweden were interviewed. Their ages ranged from 24 to 61 years (median 36), and their work experience as EMCCOs ranged from 1 to 16 years (median 4). Two participants had higher education, while the others had high school education. All participants had received a 12-week work-related training program via the employer at the start of their employment.

**Data collection/procedure**

The study was approved by the Swedish Ethical Review Authority (Dnr xxxxx) and by managers at the national response centre. The employer emailed information about the study to its employees, and those who were interested in participating contacted the researchers, obtaining written informed consent from participants. The researcher then arranged digital meetings for online interviews with the participants. Data collection was conducted by the first (JA) and last (JM) authors through semi-structured interviews that utilised an interview guide developed explicitly for this study (see Supplementary material). No pilot interview was conducted; however, the interview guide was evaluated during the first interview, which did not result in any changes. No questions were given in advance to the participants, and no questions were added to the interview guide during data collection. JA and JM had no relationship or previous knowledge of the participants. The interviews were originally conducted for this study and have never been published elsewhere. To make participants more comfortable, the interviews began with some small talk before the questions from the interview guide were asked. The questions asked included: “Can you tell us about a situation in your work when you felt stressed?”, and “Can you tell us about a situation in your work when you felt satisfied?”. Follow-up questions included: “What happened?”, “What were your thoughts?”, “How did it feel?”, and “How did you deal with it?”. These latter were asked to deepen the narratives of the participants. The participants were encouraged to provide detailed answers, drawing on their experiences in the context of each question. Data collection continued until information power (22) was assessed to be reached. The assessment of data was based on data quality and specificity. The interviews were audio-recorded, transcribed verbatim, and translated into English after the analysis. The interviews ranged from 37-83 minutes (mean 53).

**Analysis**

Qualitative content analysis was used for the study (21). Two of the authors have extensive experience with qualitative research. All authors engaged in the analysis. Three authors had experience in prehospital ambulance care, and one had previous experience in psychiatric care. All authors have work-related experiences of contact with EMCCOs, but no personal experience of working at EMCC, nor personal relationships with the participants. The analysis was performed manually without any software used. The process of analysis followed the description by Graneheim and Lundman (21). The authors read the interviews, and meaning units that responded to the aim were identified. The meaning units were condensed, and each condensed unit was labelled with a code. This was conducted by the second and third authors, who then sorted codes with similar content into groups. All authors worked on the process of further analysis. Code groups were abstracted and interpreted into subthemes, and subthemes with similar content were grouped, abstracted, and interpreted into themes. The process involved oscillating between the analysis and the raw data, reflection and discussions within the research group before finalising the results. The themes are strengthened by quotations.

**Result**

The analysis's results consisted of 12 subthemes, four themes, and one main theme (see Table 1).

Table 1. Subthemes, themes, and main theme.

| Subtheme | Theme | Main theme |
| --- | --- | --- |
| Engaging in complex calls  Dealing with demanding situations  Lacking support | Facing challenges | Having oneself as a stake |
| Managing stress symptoms  Suffering from a troubled conscience  Trying to recover | Being affected as a person |  |
| Struggling with insufficient systems and tools  Having varying trust in management  Struggling with unfavourable work conditions | Feeling ignored by organisational conditions |  |
| Valuing education and skills  Feeling strengthened by colleagues  Feeling satisfied by helping others | Drawing strength from rewarding conditions |  |

**Facing challenges**

This theme concerns engaging in complex conversations, dealing with demanding situations, and lacking support.

***Engaging in complex calls***

The participants all felt that calls relating to poor mental health and suicide threats were some of the most demanding scenarios. By quickly connecting with people who felt overwhelmed to the point of not wanting to live any more, they aimed to prevent and redirect potential suicidal actions.

*A case where a younger guy calls. He first says to me: I apologise that you are the last person I’ll talk to. So, he has cut himself very, very severely, and he’s bleeding to death and wants to talk to someone while he bleeds out. (P 16)*

The participants compared calls with people with poor mental health to cardiac arrests, stating that they preferred the latter because they were concrete, and it was possible to follow an algorithm. With poor mental health, there is no template to follow, and such calls cannot be rushed, as this may provoke an unwanted reaction. Calls involving children were also described as challenging, whether the child was directly impacted or the EMCCO was speaking to a child calling about an affected relative. The complexity faced by the participants, in terms of managing their emotions, directing units to the scene, and obtaining adequate information from a sad child or panicked parent, was felt demanding. These calls were perceived as difficult or traumatic, often lingering in memory. The participants described encountering harsh attitudes and being told that they were stupid, incompetent, and criminal, along with derogatory terms such as ‘whore’, by callers.

***Dealing with demanding situations***

The participants described myriad scenarios, ranging from hazardous situations with unclear circumstances to engaging with potentially dangerous individuals. A common task for an EMCCO is swiftly determining the caller’s location. Participants described situations where callers found themselves in remote or roadless areas, unable to provide precise location details. When automatic identification failed, the participants’ abilities, such as local knowledge, were often tested.

*You feel a bit powerless when you can’t find where the person is or the person doesn’t want to tell, because then we can’t send help. We can send help, but we don’t really know where. (P 8)*

***Lacking support***

The participants described receiving calls from victims of and witnesses to events with tragic outcomes and fatalities. However, the participants did not witness these scenes firsthand. When emergency units arrive at the accident site and encounter patients, they operate in a structured manner. In addition, in the aftermath of extraordinary and/or traumatic events, there is an emphasis on feedback and debriefing. These practices are crucial for the well-being of emergency personnel, who require such support. In contrast, the participants described emergency call centres as often lacking a structured system for providing feedback, which impacted the processing of events and decision-making. Some participants resorted to seeking feedback through informal channels, relying on colleagues when time allowed. However, these alternative methods often came at the cost of leisure time and restricted their everyday lives. For some participants, the absence of feedback was more pronounced; once the caller had hung up, they were not informed about the incident’s outcome. The uncertainty about how things unfolded weighed heavily on their minds, causing stress and sleepless nights. The need for timely and constructive feedback was described as crucial for both professional development and mental well-being in such a high-pressure role:

*It’s a tragic death, and then the emergency services, ambulance personnel, and everyone who has been involved gather together, and they get to process. But we don’t get to process it, which is the missing tool. (P 6)*

**Being affected as a person**

The participants described the impact of their work on their personal and private lives, which encompassed managing stress symptoms, addressing a troubled conscience, and attempting to recover.

***Managing stress symptoms***

The participants described working under stress, which led to various consequences, including a poor work atmosphere, constant stress, and feelings of failure and inadequacy. They discussed how stress manifested in physical and mental symptoms, such as headaches and heart palpitations. Additionally, they mentioned experiencing anxiety during calls and even afterwards, especially when adrenaline surged during critical moments, and they needed to complete key tasks.

*At that moment, it was just a matter of going for it and solving the situation, but afterwards, I felt terrible. I was so stressed that my t-shirt was shaking due to my heart pounding. (P 3)*

The participants described feeling numb to the constant alarm, suppressing feelings of stress, and maintaining a façade to avoid thinking about and prioritising their well-being. They struggled with fatigue, fragmented focus, and loss of motivation and job satisfaction. Some struggled to cope with the demanding work conditions and considered resigning, despite their genuine fondness for their work.

***Suffering from a troubled conscience***

The participants described callers who were stressed, shouting, swearing, and demanding an ambulance immediately because someone was about to die. They stated that the dispatch of ambulances could be delayed when callers cannot answer questions quickly enough. This fostered a troubled conscience in the aftermath of situations in which an ambulance was not sent immediately, even though it would have been necessary, and such an action would have deviated from guidelines. The participants described situations in which their troubled consciences were sources of stress, for example, not knowing what happened to a caller they had helped during a long call or wondering if a situation could have been resolved better. Sometimes, the person seeking help calls the emergency services again to ask why help has been delayed, which also causes an increase in stress.

*They wait a long time, it’s not connected to my effort, but it ends up on my conscience because they call back and wonder where we are. (P 15)*

***Trying to recover***

The participants described work-related stress seeping into their personal lives, affecting their relationships. They found it hard to muster the energy to answer calls from parents or friends, and some even stopped keeping up with the news during their free time, losing touch with the outside world. The strain drained them, prompting them to hibernate at home during holidays, seeking rest and rejuvenation until they returned to work. The participants described various strategies to cope with stress during their downtime, including running, spending quality time with friends, and seeking solace and release. Others withdrew, becoming less sociable and turning inwards. The fear of being called in to work on their days off loomed over them, leaving little room for planning leisure activities.

*“We often get chased in our spare time. I received a text a while ago stating that a lot of staff are missing for the weekend, and since I'm scheduled, I know how stressful it’s going to be.” (13).*

**Feeling ignored by organisational conditions**

This theme pertains to workplace conditions, including struggling with insufficient systems and tools, having varying trust in management, and struggling with unfavourable work conditions.

***Struggling with insufficient systems and tools***

Errors and deficiencies in various technical systems and work tools were reported to cause stress and frustration for the participants, particularly during critical calls. These issues could arise from technology freezing, perceived inefficiencies in systems, and concerns that system failures might affect callers. The participants highlighted a new tool designed to facilitate rapid triage, but perceived it as an impediment to efficient assessment. The participants who worked as EMCCOs before the tool's implementation instead relied on their established routines, as they felt that the tool was challenging for assessment and frustrating for callers.

*They shouldn’t suffer because we are dealing with this system. Instead, sometimes I have noticed that this is a priority one case with severe chest pain radiating out, so I dispatch the ambulance first and then write down all the questions, to make it go quickly, of course. (P 2).*

***Having varying trust in management***

The participants felt that management does not actively work to ensure that the perspectives and emotions of EMCCOs are respected, and that they can express their feelings about situations. Further, they reported that colleagues had left their jobs due to a lack of confidence in management. They expressed powerlessness to influence their work situations and felt their concerns were not addressed. The participants described working conditions that seemed unsustainable, personnel policies that were felt to be inadequate, fear of repercussions for processing errors, minute-by-minute monitoring, and top-down management. According to the participants, these factors contribute to stress and a sense of underappreciation. They highlighted the value of receiving support from and being heard by their managers, even though many remained uncertain about their ability to impact their overall work situation.

*The possibility of influencing my work is practically zero. I feel incredibly tied down. I don’t know how I can influence the work. It doesn’t matter what suggestions you make or what you point out, it’s just… The opportunities don’t exist. (P 6)*

***Struggling with unfavourable work conditions***

The participants described working overtime, feeling overloaded, and the difficulties of shift work and staff shortages. They faced a relentless stream of calls, leading to ever-increasing queues and unnecessary referrals. Sick leave and cancelled breaks exacerbated the situation. Additionally, a tense atmosphere sometimes prevailed among colleagues and was even contributed to by managers. According to the participants, some managers sometimes said those who struggled were not suited to the job. The participants expressed dissatisfaction with their salaries, feeling their compensation did not match their efforts. Furthermore, the rigid schedule left little room for individual preferences. In critical incidents, multiple calls could revolve around the same case, such as a crash with numerous victims, resulting in a heavy workload and extensive call queues.

*But some days are very, very tough, and you are very, very tired. And there are a lot of emotions. You don’t really have time to think it through. You just sit there and become a bit like a machine some days. And then I think, is it worth it for me? And how could I manage it? But at the same time, I want to do it. My thought is that I want to keep doing this. (P 5)*

**Drawing strength from rewarding conditions**

The participants described valuing education and skills, feeling strengthened by colleagues, and feeling satisfied by helping others.

***Valuing education and skills***

The participants were varied in terms of their knowledge and level of education; many lacked relevant higher education and experience in healthcare professions. Those with prior experience, such as from healthcare or rescue services, felt more confident in their roles as EMCCOs due to this prior knowledge. The participants described a sense of pride in their skills and expertise, and a satisfaction in using their education to help people in need. However, they also described difficulties in assessing various situations over the phone. They felt there were continuous training opportunities, but these had to be pursued outside of working hours due to staff shortage. They described a dissatisfaction with insufficient competence and a lack of support from management in developing knowledge.

*We have a new rescue organisation, so to learn about it, I have to work a whole overtime day tomorrow; otherwise, I won’t receive the training. So yes, we are not given the opportunity to do things, so all that has to be done in our own time if we want to do it. (P 7).*

***Feeling strengthened by colleagues***

The participants all felt that their colleagues played a crucial role as a source of support, assistance, and relief. A positive workplace atmosphere was essential, ensuring they felt safe and comfortable with their closest colleagues at the alarm centre. Beyond their immediate team, relationships with other colleagues, such as ambulance staff, were supposed to be equally significant. Collaboration was felt to foster team spirit and mutual understanding. However, friction occasionally arose due to differing perspectives regarding work situations.

*There are ambulance stations where you know that the whole station will start shouting as soon as they get an alarm because they disagree. To avoid it, we have been to their staff meetings to explain these rules and why and what the region has ordered, and they have refused to understand. (P 7)*

***Feeling satisfied by helping others***

The participants found satisfaction in resolving situations and dealing with diverse tasks rapidly, akin to piecing together a complex puzzle. They relished the ebb and flow of tasks, appreciating the dynamic nature of their role. Making a tangible impact on callers and receiving gratitude for solving cases and handling calls held significant importance. The job represented a genuine opportunity for the participants to provide real assistance; this sense of purpose brought fulfilment. However, despite the ability to effect change, many of the participants described colleagues who opted to leave their employment after a brief stint due to its unsustainability:

*Then there’s the salary, of course. It’s not particularly good. This is more of a calling. Since I’m not a healthcare person, I see it this way that I still have the opportunity to help and save lives is a gift. (P 2)*

**Discussion**

This study aimed to describe work-related psychosocial health among EMCCOs. The results were divided into one main theme, “Having oneself as a stake,” and four themes: “Facing challenges,” “Being affected as a person,” “Feeling left out by organizational conditions,” and “Drawing strength from rewarding conditions.”

The results from this study can be illuminated by the Conservation of Resources Theory. The theory underscores people’s endeavors to preserve and strengthen personal resources (23). Our results show that EMCCOs invest emotional resources in their work, which, according to the theory, means a high degree of resource use. If such use is not met by recognition and social support, it can lead to stress and a sense of exposure. At the same time, we identify rewarding resources such as education, the possibility to help others, and collegial support. These can be viewed as protective factors that could contribute to rebuilding the balance of resources. Thus, mirrored through the Conservation of Resources Theory, participants' experience of both stress and reward is linked to their access to, and loss of, key psychological and social resources. We argue that this perspective highlights the importance of strengthening access to both psychological and social resources for EMCCOs.

The results can also be illuminated by the Job demand-control-support theory (24), which focus on the balance between job demand and control over the work situation. Our results show that participants met high emotional and cognitive demands and at the same time, experienced limited support from their management. This can be interpreted as limited scope for decision making and control, a combination that, according to the theory, increases the risk for stress-related ill-health. On the other hand, the results present protective factors: collegial support and education that strengthen feelings of competence and scope for action. Combined with the possibility to help others, these factors contribute to regulating the effects of high demands, a sense of meaningfulness, and thereby, increased psychological control (24). We argue that this perspective highlights the importance of strengthening EMCCOs influence and support structures to foster a sustainable work environment.

The results highlight the importance and value of feedback following incidents and a collegial feeling. The challenges faced by EMCCOs in their daily work include talking to vulnerable people in complex situations, aggravating circumstances in their workplaces, and not being able to see an event with their own eyes. Research conducted by Linderoth et al. (25), in response to this latter obstacle, investigated whether live video could better facilitate assessment by EMCCOs, and found that video calls led to more accurate and faster assessments. This was especially helpful when the calls concerned complex situations such as sick children or unconscious patients. Previous studies underscore the challenge of communicating with distressed callers, especially in traumatic situations. EMCCOs put their well-being on the line, risking verbal aggression from agitated callers (26). Research also shows that EMCCOs are challenged daily in complex situations, such as traumatic emergency calls, following which there is often insufficient or non-existent debriefing (27). One review suggests that debriefing with staff who work in clinical settings can reduce PTSD symptoms, and subjective evidence suggests that clinical staff members perceive debriefing to be useful. However, no firm conclusions regarding the effects of debriefing have been drawn due to the limited literature (28).

The results illuminate calls about complex situations, for example calls about a suspected cardiac arrest. Responding to such a call involves many steps, which must be carried out during a highly stressful situation for the caller. EMCCOs face complex situation and must guide the caller first to confirm cardiac arrest, then start and perform CPR, while alerting the nearest emergency units to the event. However, EMCCOs described such calls as relatively uncomplicated; they confidently navigate these situations thanks to well-established algorithms and routines. Calls involving threats of suicide or mental illness was described as more challenging. Previous research suggests that the worst stressors are calls involving suicidal intent, where the location of the caller is unclear or unknown, and that involve injured children (26). Lyra et al. state that witnessing suicidal behaviour has an impact on the personal life, professional life, and mental health of responders, and can lead to emotional suffering (29). In line with our theoretical arguments, future research could explore the benefits of enhanced education and support for EMCCOs, particularly related to mental health. Strengthening EMCCOs skills in handling complex and uncertain situations could contribute to a more sustainable work environment and improve emergency call outcomes (26).

The results show that work influence the private lives of EMCCOs, including experiences of physical and mental health symptoms. This aligns with a review article highlighting the effects of shift work on physical activity during leisure time. Shift work has been associated with back problems, poor eating habits, and obesity. Additionally, insufficient breaks at work leave EMCCOs mentally and physically exhausted (27), and immense perceived pressure and no respite between taxing calls make EMCCOs vulnerable to stress (26). They struggle with fatigue, fragmented focus, and loss of motivation and job satisfaction. One study examined mental health in relation to employees' work environment in different companies. The results indicated that those who felt most unwell were the ones who worked longest without a break, were most exposed to hazards in their work, faced more challenging work demands, and had less opportunity to influence their work situation compared to those who worked fewer hours per week, had lower demands from employers, and enjoyed a greater degree of autonomy in their work (29).

The results highlight that the daily influx of alarm calls poses a challenge for EMCCOs. They grapple with unpredictable and demanding work, all while safeguarding their own work-related psychosocial health after handling critical and complex cases. Participants lamented the lack of managerial support and their inability to influence their work environment. Sadly, this echoes findings from other studies, where suggestions for improvement made by EMCCOs often fall on deaf ears within management circles. Underscored by both the Conservation of Resources Theory and the Job demand- Control-Support theory, there is a risk that negative stress and poor psychosocial health will escalate where there is a lack of resources and support from management at an organisational level. This raises concerns about the work situation of EMCCOs (6, 27).

**Strength and limitations**

Aspects of trustworthiness were considered throughout the study process (30). Participants varied in gender, age, and working years, which strengthens the findings in the study. The participants described stressful situations, and the results show that they are under strain. Some might argue that the results, therefore preferably report on negative aspects of psychosocial health. Nevertheless, the purpose of the study was to highlight work-related psychosocial health, and the interview guide included questions that shed light on both challenges and satisfactions. We consider it a strength that the participants had experience of both. The authors had no pre-understandings of the study context. Therefore, to ensure relevant interpretations, the authors moved back and forth between their interpretations and the original data. For credibility, quotations from several different interviews are shown to exemplify the interpretations. For transferability, participants, context, and data-collection and analysis processes are described thoroughly. The participants shared various experiences of emergency calls and encounters with people with diverse needs. This ensured rich and varied data, which is information power.

**Conclusion**

Most of the findings in this article present a negative view of the work-related psychosocial health of EMCCOs, which in turn poses a risk of deteriorating the work environment. Poor working conditions for EMCCOs can complicate their tasks, making it less straightforward to assess the severity of emergency calls. When emergency calls lose effectiveness and response times increase, the consequences ripple through society. From a societal perspective, declining service quality can lead to increased costs, more serious incidents, accidents, and poor health. Moreover, a subpar work environment can result in ill health and increased sick leave among EMCCOs, adding further disadvantages and costs. This highlights the need for management to work with continuous improvements in the work environment and psychosocial health of EMCCOs. We suggest that managers focus on the employees' work-related psychosocial health, while the EMCCOs focus on the health and safety of the callers. Emergency service management is suggested to extend the induction training period with more education on crisis reaction for EMCCOs before employment. The results of this study can serve as a foundation for future research and efforts to enhance the work environment of EMCCOs.

**Declarations**

**Ethical approval and consent to participate.**

The sensitive subject of psychosocial health warranted that ethical issues be considered. In line with Swedish law on research ethics and sensitive data, this study received approval from the Swedish Ethical Review Authority (Dnr 2021 05427-01). Ethical issues were considered throughout the process in line with the Declaration of Helsinki (31). The participants received written information via e-mail about the purpose of the study. All participants provided written informed consent. They were informed that participation was voluntary and that they could withdraw without stating a reason. The interviews were treated confidentially and anonymised before the analysis.

**Consent for publication**

Not applicable.

**Availability of data and materials**

The datasets used and/or analysed during the study are available from the corresponding author on reasonable request.

**Competing interests**

The authors declare that they have no competing interests.

**Funding**

No funding.

**Author contributions**

JA and JM were responsible for designing the study and engaged in conceptualization, formal analysis, investigation, methodology, project administration, resources, visualization, writing the original draft, including review and editing the manuscript. EG and MN engaged in formal analysis and writing the original draft, supervised by JA. All authors approved the final manuscript.

**Acknowledgements**

The authors would like to thank the EMCCOs who participated in the study.

References

1. Kulczycka, K., Chilimoniuk, B., Rymuszka, A., Stychno, E., Bałanda-Bałdyga, A., & Pałucka, K. (2025). Cortisol levels and perceived stress in emergency call operators. Frontiers in Public Health, 13, 1539516.

2. Osório, C., Talwar, S., Stevelink, S. A. M., Sihre, H. K., Lamb, D., & Billings, J. (2025). Systematic review and meta-analysis on the mental health of emergency and urgent call-handlers and dispatchers. Occupational Medicine, 75(6), 282-291.

3. Roivainen P, Hoikka MJ, Raatiniemi L, Silfvast T, Ala-Kokko T, Kääriäinen M. Telephone triage performed by nurses reduces non-urgent ambulance missions: a prospective observational pilot study in Finland. Acta Anaesthesiol Scand. 2020;64:556–63.

4. Al-Shaqsi S. Models of international emergency medical service (EMS) systems. Oman medical journal. 2010;25(4):320.

5. Klimley KE, Van Hasselt VB, Stripling AM. Posttraumatic stress disorder in police, firefighters, and emergency dispatchers. Aggression and violent behavior. 2018;43:33-44.

6. Leonardsen A-C, Ramsdal H, Olasveengen TM, Steen-Hansen JE, Westmark F, Hansen AE, Hardeland C. Exploring individual and work organizational peculiarities of working in emergency medical communication centers in Norway-a qualitative study. BMC Health Services Research. 2019;19:1-9.

7. Blalock JR, Black RA, Bourke ML, Van Hasselt VB. Emergency Communication Operators: Findings from the National Wellness Survey for Public Safety Personnel. Journal of Police and Criminal Psychology. 2024;39(1):34-43.

8. Holmström IK, Kaminsky E, Lindberg Y, Spangler D, Winblad U. The perspectives of Swedish registered nurses about managing difficult calls to emergency medical dispatch centres: a qualitative descriptive study. BMC nursing. 2021;20:1-8.

9. Wagner A, Tsarouha E, Ög E, Preiser C, Rieger MA, Rind E. Work-related psychosocial demands related to work organization in small sized companies (SMEs) providing health-oriented services in Germany–a qualitative analysis. BMC Public Health. 2022;22(1):390.

10. Schreibauer EC, Hippler M, Burgess S, Rieger MA, Rind E. Work-related psychosocial stress in small and medium-sized enterprises: an integrative review. International journal of environmental research and public health. 2020;17(20):7446.

11. Lilly MM, London MJ, Mercer MC. Predictors of obesity and physical health complaints among 911 telecommunicators. Safety and health at work. 2016;7(1):55-62.

12. Nowrouzi-Kia B, Nadesar N, Casole J. Systematic review: Factors related to injuries in small-and medium-sized enterprises. International journal of critical illness and injury science. 2019;9(2):57-63.

13. Perez RA, Jetelina KK, Gonzalez JMR. The chronic health effects of work-related stressors experienced by police communications workers. Safety and health at work. 2021;12(3):365-9.

14. Vanderloop L. Sustainable Coping Among Experienced 911 Operators: The University of Western Ontario (Canada); 2021.

15. Wojciechowska M, Jasielska A, Ziarko M, Sieński M, Różewicki M. Mediating role of stress at work in the relationship of alexithymia and PTSD among emergency call operators. International journal of environmental research and public health. 2021;18(23):12830.

16. Steinkopf B, Reddin RA, Black RA, Van Hasselt VB, Couwels J. Assessment of stress and resiliency in emergency dispatchers. Journal of police and criminal Psychology. 2018;33(4):398-411.

17. Haugen PT, McCrillis AM, Smid GE, Nijdam MJ. Mental health stigma and barriers to mental health care for first responders: A systematic review and meta-analysis. Journal of Psychiatric Research. 2017;94:218-29.

18. Spjeldnæs TB, Nilsen KAV, Myrmel L, Sørnes J-O, Brattebø G. “Calling for help: I need you to listen”-A qualitative study of callers’ experience of calls to the emergency medical communication centre. Scandinavian Journal of Trauma, Resuscitation and Emergency Medicine. 2023;31(1):94.

19. Ricciardelli R, Carleton RN, Mooney T, Cramm H. “Playing the system”: Structural factors potentiating mental health stigma, challenging awareness, and creating barriers to care for Canadian public safety personnel. Health. 2020;24(3):259-78.

20. McAleavy T, Choi B, Arlikatti S. Faceless heroes: A content analysis of determinants for the recruitment, career development & retention of Emergency Medical Dispatchers in the United States of America. International Journal of Disaster Risk Reduction. 2021;61:102335.

21. Graneheim UH, Lundman B. Qualitative content analysis in nursing research: concepts, procedures and measures to achieve trustworthiness. Nurse education today. 2004;24(2):105-12.

22. Malterud, K., Siersma, V. D., & Guassora, A. D. (2016). Sample size in qualitative interview studies: guided by information power. Qualitative health research, 26(13), 1753-1760.

23. Hobfoll, S. E. (1989). Conservation of resources: a new attempt at conceptualizing stress. American psychologist, 44(3), 513.

24. Karasek R, Theorell T. Healthy work: stress, productivity, and the reconstruction of working life. New York: Basic Books; 1990.

25. Linderoth G, Lippert F, Østergaard D, Ersbøll AK, Meyhoff CS, Folke F, Christensen HC. Live video from bystanders’ smartphones to medical dispatchers in real emergencies. BMC Emergency Medicine. 2021;21:1-10.

26. Říha R, Hůla M. Work-Related Stressors Associated with Emergency Calls to the Fire and Rescue Service Operators and Police Operators of the Czech Republic. Journal of Police and Criminal Psychology. 2024;39(1):104-17.

27. Smith EC, Holmes L, Burkle FM. Exploring the physical and mental health challenges associated with emergency service call-taking and dispatching: a review of the literature. Prehospital and disaster medicine. 2019;34(6):619-24.

28. Scott Z, O'Curry S, Mastroyannopoulou K. The impact and experience of debriefing for clinical staff following traumatic events in clinical settings: A systematic review. Journal of Traumatic Stress. 2022;35(1):278-87.

29. Zeng Z, Guo Y, Lu L, Han L, Chen W, Ling L. Mental health status and work environment among workers in small-and medium-sized enterprises in Guangdong, China-a cross-sectional survey. BMC Public Health. 2014;14:1-8.

30. Alfsen D, Møller TP, Egerod I, Lippert FK. Barriers to recognition of out-of-hospital cardiac arrest during emergency medical calls: a qualitative inductive thematic analysis. Scandinavian journal of trauma, resuscitation and emergency medicine. 2015;23:1-8.

31. Møller TP, Jensen HG, Viereck S, Lippert F, Østergaaard D. Medical dispatchers’ perception of the interaction with the caller during emergency calls-a qualitative study. Scandinavian Journal of Trauma, Resuscitation and Emergency Medicine. 2021;29:1-10.

32. Speziale HS, Streubert HJ, Carpenter DR. Qualitative research in nursing: Advancing the humanistic imperative: Lippincott Williams & Wilkins; 2011.

33. Declaration of Helsinki, Ethical Principles for Medical Research Involving Human Subjects <https://www.wma.net/wp-content/uploads/2018/07/DoH-Oct2008.pdf>: The World Medical Assosiation; 2008 Assessed: December 12th 2024.

Table 1. Subthemes, themes, and main theme.

| Subtheme | Theme | Main theme |
| --- | --- | --- |
| Engaging in complex calls  Dealing with demanding situations  Lacking support | Facing challenges | Having oneself as a stake |
| Managing stress symptoms  Suffering from a troubled conscience  Trying to recover | Being affected as a person |  |
| Struggling with insufficient systems and tools  Having varying trust in management  Struggling with unfavourable work conditions | Feeling ignored by organisational conditions |  |
| Valuing education and skills  Feeling strengthened by colleagues  Feeling satisfied by helping others | Drawing strength from rewarding conditions |  |
